# Influence of manufacturing methods and use of CoCr-based abutments on vertical and horizontal marginal fit and torque loss in implant-supported prostheses

**DOI:** 10.1590/1678-7757-2024-0589

**Published:** 2025-05-30

**Authors:** João Pedro Justino DE OLIVEIRA LIMIRIO, Jéssica Marcela de Luna GOMES, Joel Ferreira SANTIAGO-JUNIOR, Cleidiel Aparecido Araújo LEMOS, Maria Cristina Rosifini Alves REZENDE, Eduardo Piza PELLIZZER

**Affiliations:** 1 Universidade Estadual Paulista “Júlio de Mesquita Filho” Faculdade de Odontologia de Araçatuba Departamento de Materiais Dentários e Prótese Araçatuba SP Brasil Universidade Estadual Paulista “Júlio de Mesquita Filho” (UNESP), Faculdade de Odontologia de Araçatuba, Departamento de Materiais Dentários e Prótese, Araçatuba, SP, Brasil.; 2 Universidade de São Paulo Faculdade de Odontologia de Ribeirão Preto Departamento de Materiais Dentários e Prótese Ribeirão Preto SP Brasil Universidade de São Paulo, Faculdade de Odontologia de Ribeirão Preto (FORP-USP), Departamento de Materiais Dentários e Prótese, Ribeirão Preto, SP, Brasil.; 3 Universidade Federal de Juiz de Fora Departamento de Odontologia Governador Valadares MG Brasil Universidade Federal de Juiz de Fora, Departamento de Odontologia, Governador Valadares, MG, Brasil.

**Keywords:** Dental Implant, CAD/CAM, Zirconia

## Abstract

**Objectives:**

This *in vitro* study evaluated implant-supported single crowns with external connections fabricated using different techniques (cast and CAD/CAM) and materials (metal-ceramic and translucent zirconia) regarding vertical and horizontal marginal fit and torque loss before and after mechanical cycling.

**Methodology:**

A total of 50 specimens prepared using cast (lost wax) and CAD/CAM were divided into five groups—MC (metal–ceramic crowns), ZrB (Co-Cr base, coping milled in zirconia and feldspathic ceramic veneering), Zr (coping milled in zirconia and feldspathic ceramic veneering), MZrB (Co-Cr base and monolithic translucent zirconia crown), and MZr (monolithic translucent zirconia crown)—and subjected to 5×10⁶ mechanical cycles at a 30° angle at 37°C, and an applied load of 150N at 2Hz. All crowns were screwed with a 30Ncm torque. Vertical and horizontal marginal misfit (measured using a 3D optical microscope) and torque loss were assessed before and after mechanical cycling. Statistical analyses used a significance value of 0.05.

**Results:**

Before cycling, MC (93.93±22.84µm) and MZrB (66.12±11.87µm) (p<0.05) crowns showed significantly higher vertical marginal misfit values than Zr (49.92±3µm) and MZr (49.76±3,9µm). ZrB (59.96±4.66µm) crowns exhibited no statistically significant difference when compared with the other groups. MC crowns presented the highest horizontal marginal misfit values before and after cycling in group comparisons (p<0.05). MZrB had the highest torque loss (10N), with a statically significant difference when compared with MZr and Zr (p<0.05). Comparison between all groups showed no significant difference regarding the effect of mechanical cycling (p>0.05).

**Conclusion:**

CAD/CAM manufacturing resulted in lower vertical and horizontal marginal misfit values, especially for monolithic translucent zirconia crowns (MZr) before and after mechanical cycling. All groups presented torque loss before and after mechanical cycling.

## Introduction

Single implant-supported prostheses are often used to reestablish function and aesthetics after tooth loss due to a high success rate with long follow-up times.^1^ Pioneered by Branemark,^2^external connection-type implants have been widely used but not without some disadvantages, including mechanical and biological complications.^3^Vertical and horizontal misfit can cause mechanical complications, including increased tension in prosthetic components, screws, and peri-implant bone.^4^ In addition to mechanical failures, biological complications like inflammation of peri-implant tissues can also occur due to colonization by microorganisms due to microgaps. Most oral bacteria are 0.2 to 1.5-μm wide and 2 to 10-μm long and can therefore easily colonize microgaps.^5-7^

Screw-retained implant-supported prostheses require a torque wrench to consistently generate the preload force.^8,9^ A more effective preloading method is to tighten the screw to the recommended force, loosen it after a few minutes, and then retighten to the required torque value between the crown/implant set.^8,9^ Some factors can influence preload decrease and consequently screw loosening, including insufficient torque, sedimentation, vibration and micromovement, excessive flexion and fatigue, inadequate implant position, occlusal design or inadequate crown anatomy, variation of the hexagon dimension, small differences in fit and accuracy, tension in the crown/implant assembly (due to poor occlusal adjustment), and inadequate screw design.^10^Restorative materials are one of the determining factors in a successful implant-supported prosthesis; thus, crown/implant interface fit can be influenced by the materials and techniques used during the manufacturing process.^11,12^Lost-wax is used to manufacture metal–ceramic crowns by casting metal alloys like chromium–cobalt which, due to various laboratory steps, can lead to complications such as misfit.^13^Prefabricated abutments with metallic strap were introduced for predictable fit to the implant platform, but present aesthetic problems, due to translucency of the ceramic coating materials, and corrosion over time which may affect the color of the peri-implant soft tissue.^14,15^

CAD/CAM (computer-aided design and computer-aided manufacturing) technology reduces clinical time by producing low-cost prosthesis with better fit. Moreover, the evolution of ceramic materials has introduced zirconia as a material with high biocompatibility, mechanical resistance, and aesthetically pleasing compared with metals. Thus, prefabricated and customized abutments and monolithic zirconia crowns have been used, which can be installed directly on the implant platform or on a metal base.^13,16,17^ Garine, et al.^18^ (2007) and Pereira, et al.^19^ (2019) reported that using zirconia directly on the implant platform can cause hexagon implant wear and consequently misfit and torque loss.

According to ISO 14801:2016,^20^mechanical cycling is the recommended test for simulating mastication factors such as occlusal load, temperature, humidity, and time of use related to clinical conditions. Thus, this *in vitro* study evaluated implant-supported single crowns with external connections fabricated using different techniques (cast and CAD/CAM) and materials regarding vertical and horizontal marginal fit and torque loss before and after mechanical cycling. We tested two null hypothesis: 1) vertical and horizontal marginal misfit would show no difference between the techniques (cast, CAD/CAM and CoCr base) and materials used for manufacturing implant-supported single crowns with external connections, before and after mechanical cycling; 2) torque loss would show no difference between the techniques (cast, CAD/CAM and CoCr base) and materials used for implant-supported single crowns with external connections, before and after mechanical cycling.

## Methodology

**Figure 1:**
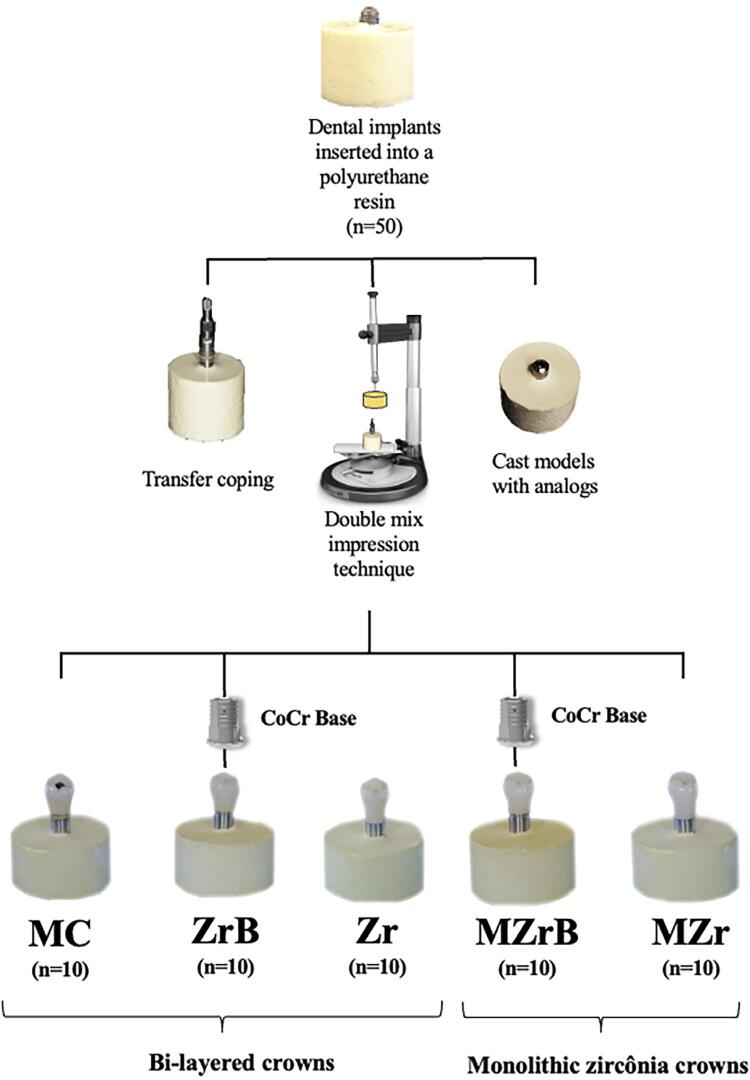
Experimental design. Bilayered ceramic crowns: metal–ceramic crown (MC), veneered zirconia crown (Zr), veneered zirconia crown with CoCr base abutment (ZrB), monolithic translucent zirconia crown with CoCr base abutment (MZrB), and monolithic translucent zirconia crown (MZr).

A total of 50 implant-supported single crowns with external connection (Ø 4 mm×11 mm) (HE EASY- GRIP Porous RD, Conexão Sistema de Prótese) were inserted into polyurethane resin (F-16 FastCast Polyurethane, Axson), ideal for biomechanical tests, ^21^ 3 mm below the implant platform (ISO 14801:2016)^20^ and divided into five groups (n=10): MC, ZrB, Zr, MZrB, and MZr (Figure 1).

Cast models (obtained by printing the dies with previously inserted implants) were scanned using an extraoral scanner (S600 ARTI, Zirkonzahn Worldwide) attached to a scan body (Conexão Sistema de Prótese). Infrastructures and crowns were modeled using Zirkonzahn Modellier software (Zirkonzahn Worldwide). Monolithic translucent crowns (MZrB and MZr) followed the anatomy of the lower second premolar. Milled infrastructures (ZrB and Zr) had smaller dimensions by 1.5 mm for later veneering ceramic application. After modeling, the digital files (STL) were exported and milled (Milling Machine M1, Zirkonzahn Worldwide) in partially yttria-stabilized zirconia (Ice Zirkonzhan Translucent, Zirkonzahn Worldwide) for copings and monolithic crowns in translucent zirconia (Prettau, Zirkonzahn Worldwide), sintered at 1600 °C (Sinterofen 300S – Zirkonzahn Worldwide) according to the manufacturer’s recommendations. Copings and veneering ceramics for MC, ZrB, and Zr, were produced by preparing split matrices using the random choice of monolithic crown and milled group coping with laboratory silicone (Zetalabor, Zhermack SpA) to standardize crown dimensions.

UCLA-Co-Cr components (Conexão Sistemas de Prótese) were screwed on plaster models with analogues (Conexão Sistemas de Prótese) and the split matrix was then positioned. The matrix was filled with liquid wax (GEO-Crowax) and, after cooling, the necessary adjustments were made. The set was sprayed with the coating material (G2 Universal Investments Talladium Inc.) and taken to the Co-Cr (Cast-Cobalt Alloy Fit Cast Cobalt Co-Cr) alloy in a heated oven, following the manufacturer’s recommendations.

The copings (Co-Cr and Zr) initially underwent surface treatment and were cleaned in an ultrasonic bath with distilled water for 5 min to remove any impurities. Before application of the veneering ceramic, they were blasted with 110-µm aluminum oxide particles (Famox, Polidental) at 0.4MPa for 20s.^22^ Layers of opaque base (Duceram Kiss, Dentsply Sirona) were applied to the metal MC copings to obscure the metal.

Veneering of feldspathic ceramics was performed with a brush and condensation was conducted using vibration. Residual water was removed with absorbent paper and sintering was conducted in an oven (Programat P300, Ivoclar Vivadent) with temperature and time according to the material and its thermal expansion coefficient (TEC).^23,24^ For Zr coping, the veneered feldsphatic ceramic (Cercom Ceram Kiss, Dentsply Sirona, York, Pennsylvania, USA) had TEC of 9.2 – 820°C, and for CoCr coping (Duceram Kiss, Dentsply Sirona (Dentsply, York, Pennsylvania, USA) a TEC of 12.8 – 920°C. Finally, a layer of glaze (IPS Ivocolor Glaze Powder, Ivoclar Vivadent) was applied to all crowns for a final shine and restoration polishing.

For groups with Co-Cr base (Conexão Sistemas de Prótese), the crowns were cemented with Relyx U200 resin cement (3M, Saint Paul). A 1000g weight was placed over a device in contact with the crowns to standardize cementation. Subsequently, photopolymerization (Bluephase, Ivoclar) was applied to each face for 20s followed by a final application for 40s, after which excess cement was removed.^25,26^

All crowns were screwed (titanium screws, Conexão Sistema de Prótese) with a torque of 30Ncm using a digital torque wrench according to the manufacturers’ recommendations (Lutron TQ8880, Lutron electronic, Taiwan). After application of an initial torque of 30Ncm and a 10min waiting time, detorque was applied to mark the loss of initial torque. Then, the confirmatory torque was applied.^9,21^Detorque assessment was performed before and after mechanical cycling as follows^21,26,27^: T0: 100x (T0-Di / T0), Tf: 100x (Tc- Df /Tc), in which T0: initial torque, Di: initial detorque, Tf: final torque, Tc: confirmation torque, Df: final detorque (post-cycling). Additionally, the screw holes in all groups were filled with Isotape (TVD) and Filtek Z-350 composite resin (3M, Saint Paul) for mechanical analysis.

Specimens were initially randomized by lot using a website (https://www.randomizer.org/). Vertical and horizontal marginal fit analyses were conducted using eight predetermined equidistant points on a device, which served as a guide to measure misfit in three-dimensional optical microscope (Quick Scope, Mitutoyo).^28^ This microscope features a digital table with 350x magnification and 1 μm precision. Measurements were performed using the QSPAK computer program (Mitutoyo). To aid in the readings, the specimens were positioned on a device that allowed the microscope beam to be positioned perpendicularly to the crown/coping/implant interface, resulting in images that made misfit analyses possible. Analyses were performed before (T0) and after mechanical cycling (Tf).

**Figure 2:**
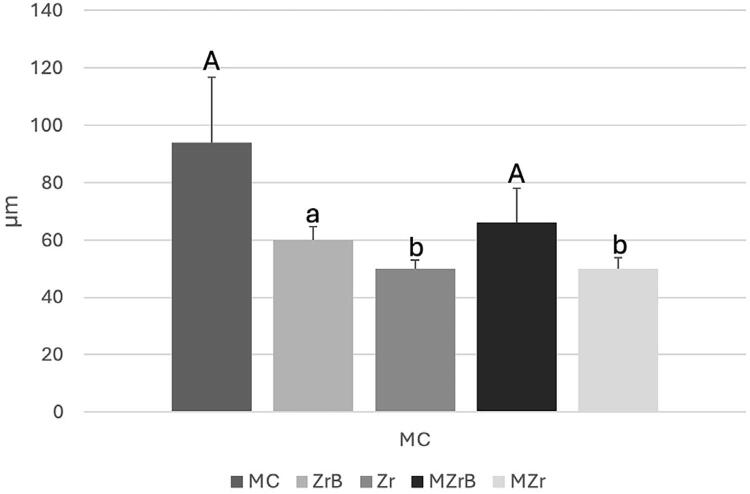
Mean (SD) initial (T0) vertical marginal misfit (µm). Different uppercase and lowercase letters (A, b): p<0.05 (MC vs. Zr, MC vs. MZr; MZrB vs. Zr, MZrB vs. MZr). Same uppercase and lowercase letters (A, a), uppercase letters (A, A), different lowercase letters (a, b): p>0.05 (ZrB vs. MC, Zr, MZrB, MZr).

Specimens were positioned on the mechanical cycling machine (Biocycle V2, BIOPDI) at a 30° angle, immersed in a tank with distilled water at 37 °C (Figure 2),^20^ and subjected to 5×10⁶ cycles (5 clinical years)^29,30^ with a 150N load applied in the center of the crown^31,32^ at 2Hz.^20^The mechanical cycling machine had an automatic device that stopped the piston given any change in the test piece, after which the number of cycles was recorded. Any cracks, chipping, or fractures in the ceramics and any screw loosening were monitored. During mechanical cycling, the crowns and crown-implant interfaces were inspected daily using a magnifying glass and, when necessary, a stereomicroscope (Discovery V20 Carl Zeiss Microscopy GmbH, Jena, Germany). Data were reported as a qualitative analysis.

Intra-examiner tests were performed on 20% of the specimens (n=10). One test was conducted before cycling and another after cycling to measure vertical and horizontal marginal misfit. The level of significance was set at .05. Systematic error (p>0.05) was calculated using paired t-test and casual error of the analyses.

Measurement data were organized into a Microsoft Office Excel table and submitted to SigmaPlot software version 12.0. All data were first analyzed using descriptive statistics. Tabulated data were then analyzed using the normality (Shapiro-Wilk test) and equality of variance tests regarding the presence or absence of failure, and difference vertical marginal misfit values. Subsequently, t-test was performed. Next, vertical and horizontal marginal misfit data were analyzed for normality distribution (Shapiro-Wilk test and equality of variance). Analysis of variance (ANOVA) was adopted to to analyze the different materials (MC, ZrB, Zr, MZrB, MZr). Normal data underwent Tukey’s post-hoc test for further analysis. For torque analysis, all data were initially analyzed by descriptive statistics using absolute data (Ncm) and percentage (%). Subsequently, data for the initial torque loss, final torque after cycling, and the difference between initial and final torque were analyzed for normality distribution (Shapiro-Wilk test and equality of variance). Kruskal-Wallis test (different material groups: MC to MZR) and Dunn’s post-test were adopted for post-test analyses.

## Results

### Qualitative analysis

In qualitative analysis, no specimens showed failure regarding loosening and/or screw fractures and 14 specimens failed during mechanical cycling with cracks on the crown: MC (5*), ZrB (3*), Zr (5*), and MZr (1*). * Number of failures per group in parentheses.

In a specific analysis of specimens that failed in each group (MC, ZrB, Zr) no significant difference was identified in relation to the level of vertical and horizontal marginal misfit (Tf-T0) between the specimens that failed and those that did not present within each group: (MC: p=.278), (ZrB: p=.990), and (Zr: p=.438).

### Vertical and horizontal marginal fit

**Figure 3:**
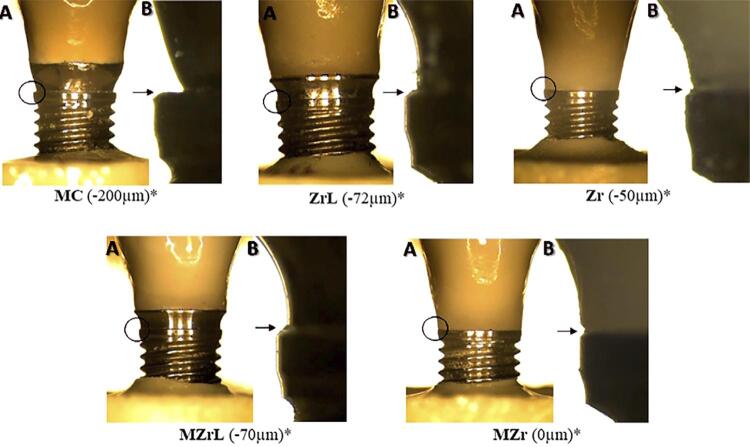
Three-dimensional optical microscope images of vertical marginal misfit in different groups. *Representative values from readings at one of the 8 points (according to the methodology). A – 50x magnification, B – 350x magnification.

MC and MZrB (p<0.05) showed significantly higher vertical marginal misfit values than Zr and Mzr in the vertical marginal misfit analysis before cycling (T0). ZrB presented no statistically significant difference when compared with the other groups (Figure 2). Figure 3 shows representative values.

**Figure 4:**
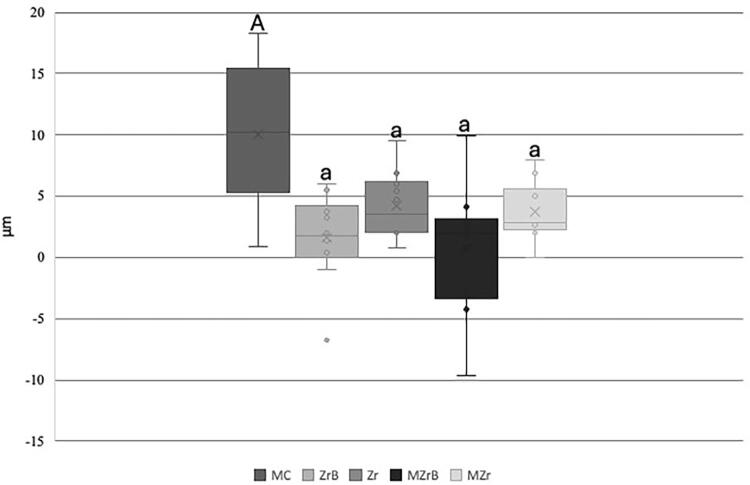
Box-Plot mean (SD) of initial (T0) and final (Tf) vertical marginal misfit (µm). Upper- and lowercase letters (A, a) show significant difference, p<0.05 (MC vs. ZrB, Zr, MZrB, MZr).

The mechanical cycling effects on vertical marginal misfit were evaluated considering the difference between the initial (T0) and final (Tf) values. MC had the highest mean difference (Tf-T0) and consequently the highest misfit after mechanical cycling compared with the other groups (p<0.05, Figure 4). Other comparisons between ZrB, Zr, MZrB, and MZr showed no difference between groups (Figure 4).

**Figure 5:**
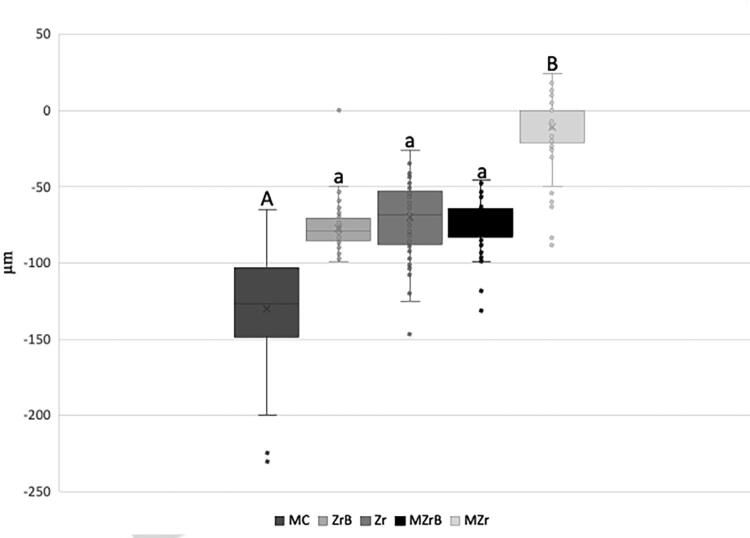
Box-plot mean (SD) of initial (T0) horizontal marginal misfit (µm). Different uppercase letters (A, B) and uppercase-lowercase combinations (A, a; B, a) indicate p<0.05, whereas same lowercase letters (a, a) indicate p>0.05. Negative values (-) represent undercontour, and positive values (+) indicate overcontour.

**Figure 6:**
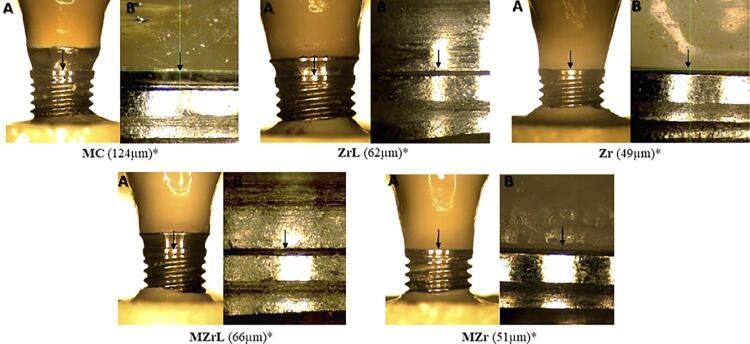
Three-dimensional optical microscope images of vertical marginal misfit in different groups. *Representative values from readings at one of the 8 points (according to the methodology). A – 50x magnification, B – 350x magnification.

According to Figure 5, horizontal marginal misfit before cycling (T0) showed a significant difference (p<0.05) with MC presenting the highest horizontal marginal misfit values, and MZr the lowest values. ZrB showed no significant difference when compared with the other groups. Figure 6 shows representative values.

**Figure 7:**
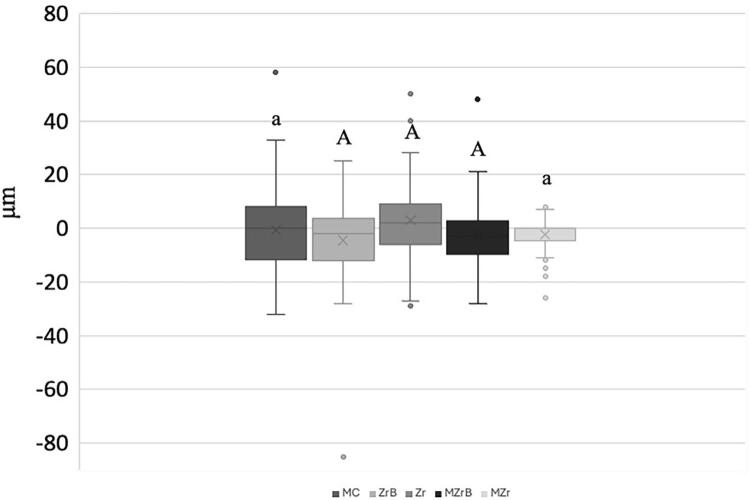
Box-Plot mean (SD) of initial (T0) and final (Tf) vertical marginal misfit (µm). Uppercase letters (A, A) indicate a significant difference (p<0.05), whereas same lowercase-uppercase letters (a, A) indicate no significant difference (p>0.05).

Mechanical cycling effects on horizontal marginal misfit according to the groups were evaluated considering the difference before (T0) and after (Tf) values. Zr showed the greatest positive mean statistically significant difference (p<0.05, Figure 7) in comparison with ZrB and MZrB. Other inter-group comparisons presented no significant difference. MZr had the lowest data dispersion, with the least variation in T0 and Tf when compared with the others (Figure 7).

### Torque loss analysis

**Figure 8:**
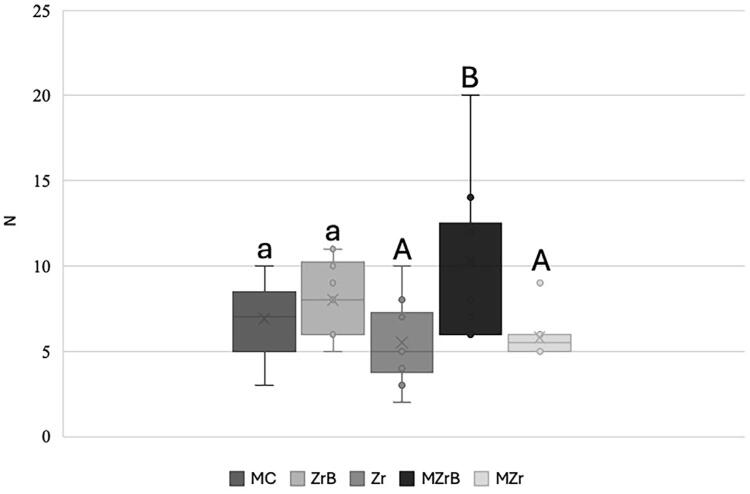
Box-Plot mean (SD) of initial (T0) torque loss. Same lowercase letters (a, a), uppercase letters (A, A), or uppercase-lowercase combinations (A, a; a, A) indicated no significant difference (p>0.05). Different uppercase letters (A, B: MZrB vs. Zr and MZrB vs. MZr) indicated a significant difference (p<0.05).

MZrB had the highest torque loss (10N) and comparison of this group with MZr and Zr was significant (p<0.05). Other comparisons showed no significant difference between the other groups. MZrB also had the highest dispersion of results and variation (Figure 8).

**Figure 9:**
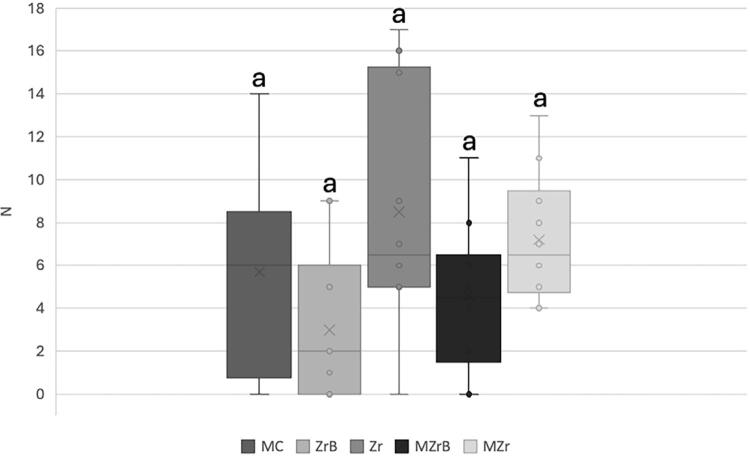
Box-plot mean (SD) of initial (T0) and final (Tf) torque loss. Same lowercase letters (a, a) indicate no significant difference (p>0.05).

As for mechanical cycling analysis, the difference in torque loss (T0-Tf) indicated higher values for the Zr group. However, we found no significant difference in the comparison between all groups (p>0.05), as shown in Figure 9.

## Discussion

Analysis results showed significant differences in vertical and horizontal marginal misfit, thus rejecting the first null hypothesis. Importantly, the success of metal–ceramic implant-supported (MC) prostheses with long follow-up times is widely reported in the literature^3,17^; however, some studies show that one of the main problems associated with cast manufacturing is the various laboratory processes involved. During casting, for example, the high temperatures lead to distortions despite the use of prefabricated Co-Cr copings (UCLA), resulting in greater vertical marginal misfit.^13,33^Additionally, a systematic review evaluating the marginal misfit of implant-supported prostheses produced by CAD/CAM compared with casting found the digital method to be superior in unitary implant-supported prostheses.^4^ These findings corroborate our results since even after undergoing a long mechanical cycling (5,000,000 cycles), the CAD/CAM-manufactured prostheses showed better vertical marginal misfit values.

Copings of the veneering (Zr) and monolithic (Mzr) crowns showed better vertical marginal misfit values, thus corroborating the advantages of this system reported in the literature such as reduced clinical time, lower cost, patient comfort, and prosthesis accuracy.^16,17^This technology provides a manufacturing virtual library containing the design of each implant or prosthetic component that aids in synthesizing structures for a more precise fit into the implant platform.^4^

The veneered ceramic (ZrB) and monolithic (MZrB) crowns cemented over prefabricated Co-Cr bases showed favorable marginal misfit values for this manufacturing method compared with casting.^13^ The negative vertical marginal misfit (intrusion) data found in these groups may be related to a microgap present before mechanical cycling, when the crown was seated on the platform.^21^

Results found higher horizontal marginal misfit values for MC (cast technique) and lower horizontal marginal misfit values for MZr (CAD/CAM), with less data dispersion, when compared with the other groups as reported in the literature.^4,34^ Importantly, immediately after the casting process in MC, which is already a factor for misfit, the pieces undergo demarcation refining of the metallic belt and polishing to render the surface smooth and shiny which contribute to it having a higher negative horizontal marginal misfit, indicating a subcontour.

Despite significant differences in horizontal marginal misfit values before and after mechanical cycling, results showed that compared with ZrB and MzrB, Zr presented misfit values close to those of the causal Dahlberg error of 2.49 µm, indicating that the difference in measured values is explained by examiner calibration rather than an actual difference between groups. However, MZr showed less variation (T0-Tf) and data dispersion.

Regarding torque loss, results indicated significant differences between the groups, as reported by some studies,^9,10,35^since geometric morphology, material used, and the manufacturing method influence screw preload. This finding rejected the second null hypothesis.

All groups showed initial torque loss (preload), especially MZrB. Some studies^36,37^reported that both the internal implant and screw threads are not completely machined smooth, which decreases the micro roughness of all metal surfaces due to contact. Hence 2% to 10% of the initial preload can be lost differing from our findings, in which the initial torque loss (preload) percentages were higher. Nonetheless, no group presented a significant difference when compared with the control group (MC). Encrustation or sedimentation relaxation values depend on the number of rough spots on the contact surfaces, implant hardness, screw surface, and load applied to the system.

CoCr bases are used as an alternative and research^12,19,38^reports that zirconia, when applied directly on the implant, causes wear and mechanical complications. Similar studies^39,40^compared prefabricated titanium abutments with zirconia abutment manufactured by CAD/CAM, showing that the latter was inferior to the former regarding fit, degree of freedom rotation, and torque loss. Hence, the latter would be greatly subjected to abutment/implant set instability, as with the crown/implant set in the present study. Applying the zirconia crown/coping directly onto the implant can produce higher screw friction when installed and consequently less torque initial loss. However, mechanical properties such as the low flexural modulus with direct screw/zirconia contact can cause higher torque loss under external forces like mechanical cycling.^9^

Single implant-supported prostheses tend to concentrate more tension on the screw thereby increasing the chances of loosening.^16^After mechanical cycling, the average contained preload percentage decreased in all the experimental groups, although the screw loosening could not be detected as no sample presented this type of failure. Since the removal torque value measures the remaining preload in the crown/screw set,^41^the torque decrease observed aligns with the screw set failure mechanism described by Bickford^42^in which the external forces gradually decrease the preload due to micro vibrations in the screw, thereby causing it to loosen.

All veneered ceramic crowns (MC, ZrB, and Zr) failed due to chipping of the veneering ceramic and monolithic crown (MZr). However, this prosthetic complication had no influence on the vertical and horizontal marginal misfit values or torque loss. Despite the different acceptable vertical marginal misfit values reported in the literature, the most used reference value is 120 µm.^43-45^ As all groups evaluated here were below this acceptance limit, all types of implant-supported prostheses studied could be viable in rehabilitation. Additionally, studies showed that the main mechanical complications for the types of prosthesis analyzed are screw loosening and veneering ceramic chipping when compared with internal connections.^3,16,46^ However, results showed that no specimen presented failures due to screw loosening, which may be explained by torque performed according to the manufacturer’s recommendation. Some limitations are inherent to the *in vitro* design, such as difficulties in simulating the stomatognathic system.

## Conclusion

- Within the limitations of this *in vitro* study, we can conclude that:
- Implant-supported screw-retained prostheses prepared by casting (metal-ceramics) presented the higher vertical and horizontal marginal misfit before and after mechanical cycling.
- Use of CoCr base is a viable alternative.
- CAD/CAM manufacturing resulted in lower vertical and horizontal marginal misfit values, especially for monolithic translucent zirconia crowns (MZr) before and after mechanical cycling.
- Although all groups presented torque loss before and after mechanical cycling, preload values successfully maintained the crown-implant union without failures due to screw loosening regardless of manufacturing technique and material used.
